# Circadian Rhythm of Heart Rate and Heart Rate Variability in Pregnancy

**DOI:** 10.21203/rs.3.rs-6075260/v1

**Published:** 2025-05-19

**Authors:** Mahkameh Rasouli, Mohammad Feli, Iman Azimi, Shahab Haghayegh, Fatemeh Sarhaddi, Hannakaisa Niela-Vilen, Anna Axelin, Pasi Liljeberg, Amir Rahmani

## Abstract

The autonomic nervous system (ANS) regulates physiological changes during pregnancy, supporting fetal development and homeostasis. Heart rate (HR) and heart rate variability (HRV) are non-invasive ANS biomarkers; however, their circadian rhythms during pregnancy remain underexplored due to the lack of continuous data collection, a gap now addressed by wearable technology. This study is the first comprehensive investigation of HR and HRV circadian rhythms throughout pregnancy using wearable devices in a free-living environment. We extract longitudinal HR and HRV from smartwatch photoplethysmography (PPG) data via a machine learning-based pipeline and employ Cosinor analysis to assess circadian rhythm characteristics. Our findings revealed significant HR and HRV circadian patterns over 16 weeks, showing a decline in HRV and an increase in HR rhythm-adjusted mean, as well as elevated nighttime stress linked to sleep disturbances and daytime fatigue. These results offer valuable insights into ANS regulation during pregnancy and highlight potential biomarkers for pregnancy complications.

## Introduction

Pregnancy is a critical period during which the autonomic nervous system (ANS) plays a crucial role in regulating biological functions and maintaining homeostasis to support fetal development [1, 2]. Evaluating ANS function through the changing patterns of heart rate (HR) and heart rate variability (HRV), widely recognized as a non-invasive indicator of ANS activity [3], offers valuable insights into the physiological adaptations occurring during pregnancy. The patterns of change in HR [4] and HRV [5] follow the circadian rhythm, which has approximately a 24-hour cycle and is among the most well-known biological rhythms [6].

Circadian rhythms are regulated by central and peripheral clocks and influenced by photic cues, such as light exposure, as well as non-photic cues, like the sleep-wake cycle, meal timing, body temperature, and physical activity [7]. Disruptions in HR and HRV circadian rhythms have been linked to various disorders [8, 9], underscoring the potential for timing-specific treatments and targeting abnormal circadian rhythms as a complementary approach to standard care practices for certain pathologies. Therefore, understanding the circadian patterns of HR and HRV during pregnancy provides valuable insights into the regulation of the ANS in response to both photic and non-photic cues and could contribute to understanding the underlying causes of pregnancy complications, such as preterm birth [10, 11], gestational diabetes [12, 13], pre-eclampsia [14, 15, 16], and eclampsia [17, 18], for which few or no strong etiological factors have been firmly established. The conceptual framework representing these relationships is illustrated in Figure 1.

There are few studies specifically investigating the circadian rhythms of HR and HRV during pregnancy. Some studies have utilized ambulatory blood pressure monitoring (ABPM) devices to capture episodic data in controlled environments, reporting only changes in HR rhythm-adjusted mean values [19, 20]. Others have employed Electrocardiogram (ECG) devices [21, 22, 23, 24], allowing for the measurement of HR and frequency domain HRV metrics. Notably, Ekholm et al. [21] conducted the first known study examining HRV circadian rhythms in pregnancy using Holter ECG, which enabled data collection during participants' normal daily activities and sleep-wake cycles. Their findings indicated that all components of HRV power spectral analysis exhibited circadian rhythms, although robust statistical evidence for presence of these rhythms was not provided. Another remarkable study involved participants in a free-living environment, adhering to strict protocols for sleep-wake cycles, diet, and physical activities [24]. This study was the first to statistically confirm the presence of circadian rhythms in HRV changes during the first trimester of pregnancy, utilizing the Cosinor method [25]. Furthuremore, studies such as those by Stein et al. [23], have not only explored the circadian rhythm of HRV but also investigated its deviations in complicated pregnancies which potentially may aid in the identification of pregnancies at risk in the early weeks.

However, a significant limitation of previous studies is the lack of long-term, continuous data collection in free-living environments. Devices like ABPM and ECG, including Holter ECG, provide only short-term and non-continuous assessments due to the challenges of prolonged lab stays or extended periods of

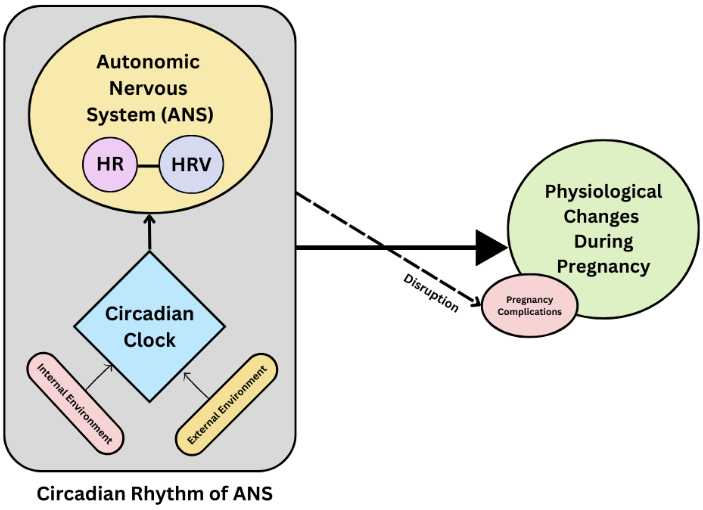

Figure 1: Conceptual Framework of Exploring HR and HRV Circadian Rhythms During Pregnancy and Their Association with Pregnancy Complications wearing these devices. This episodic approach falls short in capturing the dynamic nature of pregnancy adaptations and their circadian rhythms throughout all stages, thereby limiting our understanding of when, where, and how ANS alterations occur during pregnancy and their predictive value for pregnancy outcomes. A critical gap in the literature is the absence of studies examining circadian rhythms across all stages of pregnancy due to the lack of long-term, continuous data collection in free-living environments. While Curione et al. [24] confirmed the presence of circadian rhythms in HR and HRV changes during the first trimester, there is a notable lack of research on the existence and characteristics of circadian rhythms during the second and third trimesters, periods marked by significant physiological changes and an increased likelihood of complications.

Recent advancements in wearables for health monitoring have enabled continuous, cost-effective, and real-time data collection throughout pregnancy, including HR and HRV measurements. Devices such as Oura ring [26], Samsung Galaxy Watch [27], and Fitbit Sense [28] allow long-term monitoring in free-living settings, addressing the limitations of episodic data collection [29, 30, 31, 32, 33]. These wearables offer dual benefits of participant comfort and the ability to monitor lifestyle factors such as sleep-wake cycles, diet, and physical activities without requiring restrictive protocols. Additionally, advances in machine learning have improved the processing of wearable Photoplethysmogram (PPG) signals, an optical technique that measures blood volume changes to estimate cardiovascular metrics, enabling the extraction of reliable HR and HRV measurements. We hypothesize that this approach will support the continuous collection of HR and HRV data throughout pregnancy, potentially extending into the perinatal and postpartum phases, thereby facilitating the investigation of circadian rhythms in HR and HRV in real-world conditions.

Table 1: Participants' background information (n=30)

| Characteristic | |
|---|---|
| Ages, mean (range) | 31.5 (23–39) |
| BMI, mean (range) | 25.1 (17.8–45.9) |
| Married/cohabiting, n (%) | 30 (100) |
| Employed/full-time student, n (%) | 28 (93) |
| University level education, n (%) | 8 (27) |
| Pregnancy planned, n (%) | 27 (90) |
| Gestational age at recruitment (week+day), mean (std) | 14+2.8 (0.8+1.3) |
| Gestational age at delivery (week+day), mean (std) | 39.8+2.7 (1.3+1.9) |

This study presents the first comprehensive investigation of circadian rhythms of HR and HRV throughout pregnancy, with a particular focus on the second and third trimesters. Leveraging continuous, longitudinal data collection in a free-living setting using wearable devices, we develop machine-learning approaches to extract HR and HRV from PPG signals through a reliable processing pipeline and impute missing data. Additionally, Cosinor statistical analysis [25] is applied to assess the presence and characteristics of circadian rhythms in HR and HRV. By establishing normative patterns, this study enhances our understanding of autonomic adaptations during pregnancy, facilitates the early detection of pregnancy-related complications, and contributes to the development of personalized prenatal care strategies.

## Results

### Overview

In this study, we explored the circadian rhythms of HR and HRV in healthy pregnant women. Analyzing these rhythms provides valuable insights into the physiological adaptations during pregnancy and potential risks to maternal and fetal well-being. To achieve this, we collected continuous, longitudinal HR and HRV data from pregnant women in free-living conditions. We applied Cosinor analysis, a method specifically designed for modeling rhythmic biological processes, to interpret these patterns.

### Population Characteristics

We describe the study population to provide context for our circadian rhythm analysis results. Our study included data from 30 pregnant women monitored during their second and third trimesters. The participants were recruited at a mean gestational age of 14 weeks and followed through delivery, with a mean gestational age at delivery of 39.8 weeks. Participants had an average age of 31.5 years, ranging from 23 to 39 years. All women had low-risk pregnancy profiles, defined as having a history of full-term births (i.e., after gestational week 37) and no prior pregnancy losses. We continuously collected data using Samsung Gear Sport smartwatches [34] in free-living conditions, recording HR and HRV measurements during both sleep and daily activities. A detailed summary of population characteristics can be found in the Method section, Table 1.

### HR Circadian Rhythm

In this section, we present the results of our analysis of HR circadian rhythms during pregnancy. First, we provide the overall rhythmicity parameters for all pregnancy weeks in our study, examining trends in key rhythmicity metrics. Following this, we demonstrate a more detailed analysis of HR circadian rhythm patterns for specific pregnancy weeks.

#### Overall Rhythmicity Parameters of HR

We conducted a detailed analysis of HR circadian rhythms for all individuals across various pregnancy weeks. The Cosinor model was first applied to each participant's daily 24-hour HR data. Then, the weekly averages for each participant were calculated by averaging the daily Cosinor models across different days of the week. Finally, the population's overall HR rhythmic pattern for each week was derived as the mean of the individual participants' weekly averages.

The Cosinor analysis examined three key rhythmicity parameters: amplitude, MESOR (Midline Statistic of Rhythm), and acrophase. Amplitude refers to half the distance between the maximum and minimum values of the rhythm, indicating the intensity of fluctuations. MESOR represents the rhythm-adjusted mean around which fluctuations occur, serving as the baseline. The acrophase indicates the timing of the peak in the rhythm. Additionally, we report the p-values that assess the statistical significance of the rhythm, testing the null hypothesis that no rhythmic pattern exists in the data.

Figure 2 demonstrates the overall p-values, amplitude, MESOR, and acrophase of HR circadian rhythms for pregnancy weeks 14 to 40. As illustrated in Figure 2a, the analysis for weeks 14 to 39 shows highly significant p-values with all below the 0.01 threshold, with week 40 also significant below the 0.05 threshold, indicating significant circadian patterns in HR.

Figure 2b illustrates the amplitude of the HR circadian rhythms for weeks 14 to 40, covering the second trimester (14–27 weeks) and third trimester (27–40 weeks). As indicated, a significant downward trend is observed during the second trimester, starting at 3.5 BPM in week 14 and decreasing to less than 2 BPM by week 27. In contrast, a significant upward trend is observed in the third trimester, reaching about 3 BPM by week 40. These trends indicate that the intensity of the HR circadian rhythm decreases during the second trimester but gradually increases as pregnancy advances into the third trimester.

In addition, Figure 2c illustrates the MESOR from the Cosinor analysis of HR circadian rhythms. A significant upward trend is observed from week 14 to week 34, starting at approximately 74 BPM and rising to around 80 BPM. Following this, a highly significant downward trend emerges from week 34 to week 40, bringing the MESOR below 74 BPM by the end of the period. These findings show a shifting circadian rhythm baseline in HR, with an increase during the second trimester and a decline in the later weeks of the third pregnancy trimester.

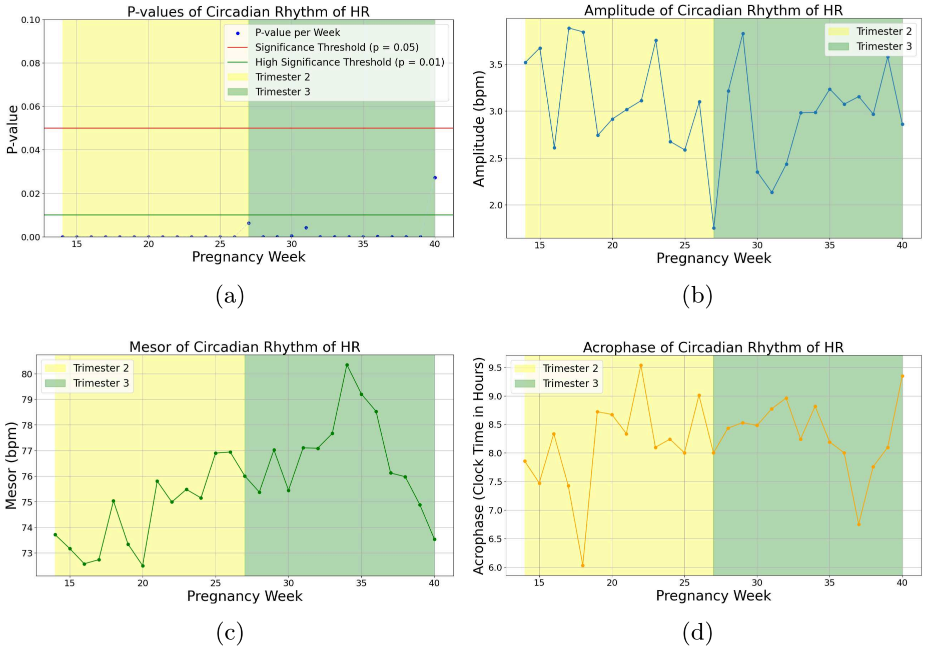

Figure 2: Cosinor analysis of HR circadian rhythms during pregnancy weeks 14 to 40, including (a) p-values, (b) amplitude, (c) MESOR, and (d) acrophase.

Figure 2d presents the acrophase of the HR circadian rhythms. As shown, no significant trend was detected in the acrophase across all weeks, indicating minimal change over time. This suggests that, overall, the timing of the peaks in HR circadian rhythms remained relatively stable throughout the pregnancy period.

### Circadian Rhythm Patterns of HR for Specific Pregnancy Weeks

We present the detailed Cosinor analysis results of HR circadian rhythms across pregnancy weeks 14, 22, 27, 30, 34, 37, and 40 (Figure 3). Weeks 14 and 40 were the first and last weeks in our analysis, while week 27 represents the end of the second trimester. Weeks 30, 34, and 37 also revealed significant findings. It should be noted that further HR circadian rhythm analyses for other pregnancy weeks are included in the Appendix (Figure 8) for reference.

In Figure 3, black dots and gray lines represent the weekly-averaged circadian data and rhythms for individual participants, respectively. The black lines also show the population-mean model, highlighting the overall rhythmic pattern for the entire population. As indicated, analysis of the population-mean HR revealed systematic changes in both timing and magnitude of daily minima and maxima across gestation. It should be noted that the number of data points (black dots) varies across weeks due to the unavailability of data from certain participants during specific weeks.

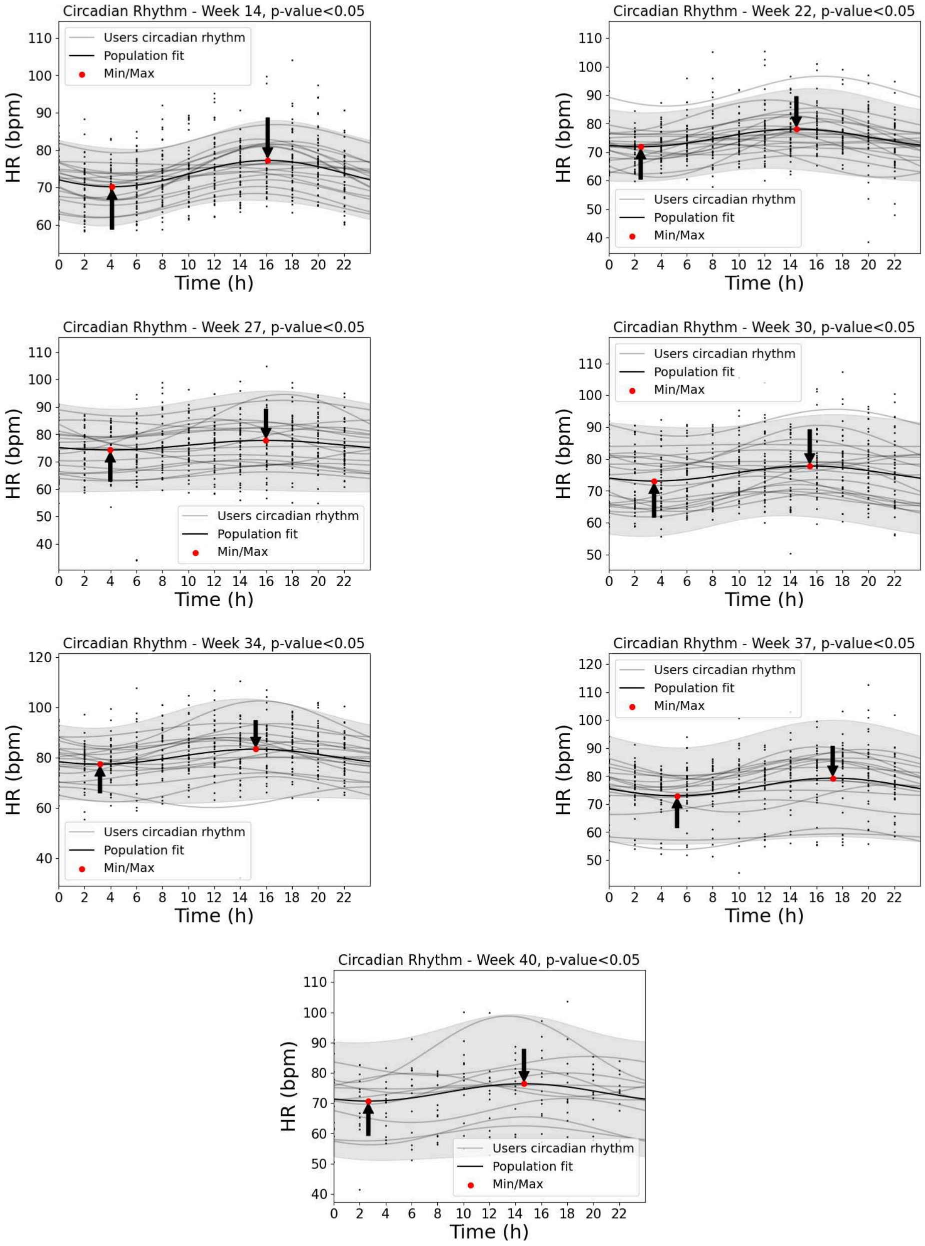

Figure 3: Cosinor analysis of the circadian rhythm of HR across pregnancy weeks 14, 22, 27, 30, 34, 37, and 40. Each subfigure shows the circadian pattern of HR for the corresponding pregnancy week. Individual participants' weekly-averaged circadian data and rhythms are represented by black dots and gray lines, respectively, while the bold black line depicts the population-mean HR circadian rhythm. It should be noted that the number of data points (black dots) varies across weeks due to the unavailability of data from certain participants during specific weeks.

In week 14, HR exhibited a minimum of around 70 bpm at 04:00 and reached its maximum of just below 80 bpm at 16:00. By week 22, both the timing and magnitude shifted, with the minimum of 70 bpm occurring earlier at 02:00 and the maximum decreasing to around 75 bpm at 14:00. At week 27, the amplitude of oscillation diminished substantially, with both minimum and maximum values converging at 75 bpm, occurring at 04:00 and 16:00 respectively. Week 30 demonstrated patterns similar to week 27, though with slightly advanced timing. A notable increase in HR values was observed at week 34, with the minimum rising to 80 bpm at 03:00 and the maximum exceeding 80 bpm at 15:00. During week 37, the timing of extrema showed the greatest delay, with minimum and maximum values (both ranging 70-80 bpm) occurring at 05:00 and 17:00, respectively. Finally, by week 40, the pattern shifted earlier again, with the minimum decreasing to 70 bpm after 02:00 and the maximum reaching around 75 bpm after 14:00.

### HRV Circadian Rhythm

This section presents the findings of our analysis of HRV circadian rhythms, focusing on the root mean square of successive differences (RMSSD) between heartbeats. RMSSD is a crucial parameter indicating the variability in time between consecutive heartbeats. We analyzed both overall HRV rhythmicity parameters throughout pregnancy and HRV circadian rhythms at specific pregnancy weeks.

#### Overall Rhythmicity Parameters of HRV

We analyzed HRV circadian rhythms for all individuals across various pregnancy weeks. Similar to our approach used for HR analysis, the Cosinor model was applied separately to each participant's daily 24-hour HRV data. Weekly averages for each participant were then calculated by averaging the daily Cosinor models across several days of the week. Finally, the population's overall HRV rhythmic pattern for each week was determined as the mean of the individual participants' weekly averages. These results, including temporal evolution of p-values, amplitude, MESOR, and acrophase across pregnancy weeks 14 to 40, are summarized in Figure 4.

As shown in Figure 4a, analysis of RMSSD demonstrated statistically significant circadian patterns ($p < 0.05$) in most pregnancy weeks. Figure 4b reveals a consistent downward trend in amplitude across both the second and third trimesters, decreasing from above 2 ms in week 14 to less than 1 ms by week 40. This trend suggests a progressive weakening of HRV rhythmic patterns as pregnancy advances.

Figure 4b demonstrates the amplitude of the RMSSD circadian rhythms throughout pregnancy. The results reveal a consistent downward pattern across both the second and third trimesters. Starting at an amplitude of above 2 ms in week 14, it declined to less than 1 ms by week 40. These findings indicate a weakening of the RMSSD rhythmic pattern as pregnancy progresses, showing a reduction in the strength of the HRV circadian fluctuations.

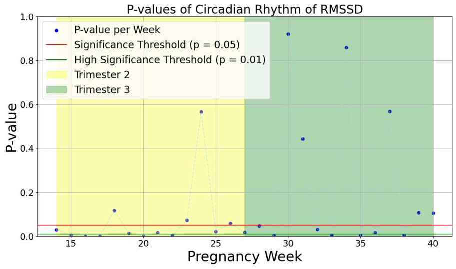

(a)

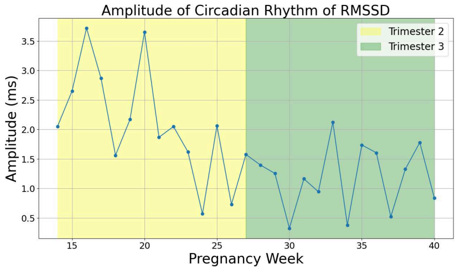

(b)

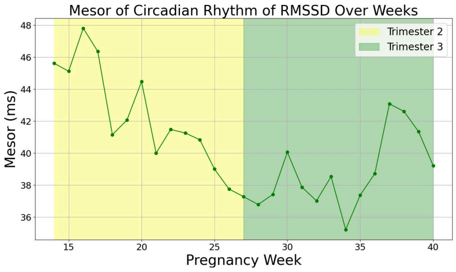

(c)

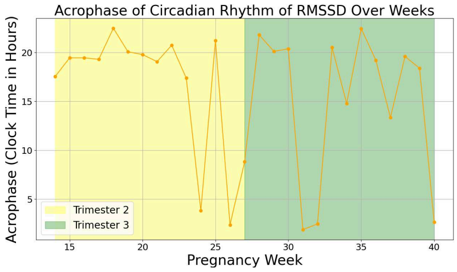

(d)

Figure 4: Cosinor analysis of HRV during pregnancy weeks 14 to 40, including (a) p-values, (b) amplitude, (c) MESOR, and (d) acrophase.

The MESOR analysis (Figure 4c) identified a significant downward trend from week 14 to week 34, with values declining from approximately 46 ms to below 36 ms. Following this decline, a transient increase was observed for MESOR during weeks 35-37, reaching approximately 43 ms, before decreasing again in the final weeks of the third trimester to around 39 ms.

As illustrated in Figure 4d, no significant trends were observed in the acrophase values of RMSSD. However, the acrophase for most pregnancy weeks occurred after 15:00, indicating that the peaks of HRV rhythms were generally concentrated in the afternoon.

#### Circadian Rhythm Patterns of HRV Across Specific Pregnancy Weeks

The results of Cosinor analysis of RMSSD for pregnancy weeks 14, 22, 27, 30, 34, 37, and 40 are demonstrated in Figure 5. The analysis revealed significant circadian variations in RMSSD for weeks 14, 22, and 27 ($p$-value $< 0.05$), while the patterns for weeks 30, 34, 37, and 40 were less significant, exhibiting a less pronounced oscillatory pattern ($p$-value $> 0.05$). Additional analyses of HRV circadian rhythms for other pregnancy weeks are provided in the Appendix (Figure 9) for further reference.

In week 14, the maximum RMSSD occurred around 6:00, with values slightly below 50 ms, while the minimum was observed just after 18:00, at approximately 45 ms. By week 22, the maximum RMSSD shifted to around 3:00 AM, exceeding 40 ms, and the minimum appeared just after 15:00, reaching about 40 ms.

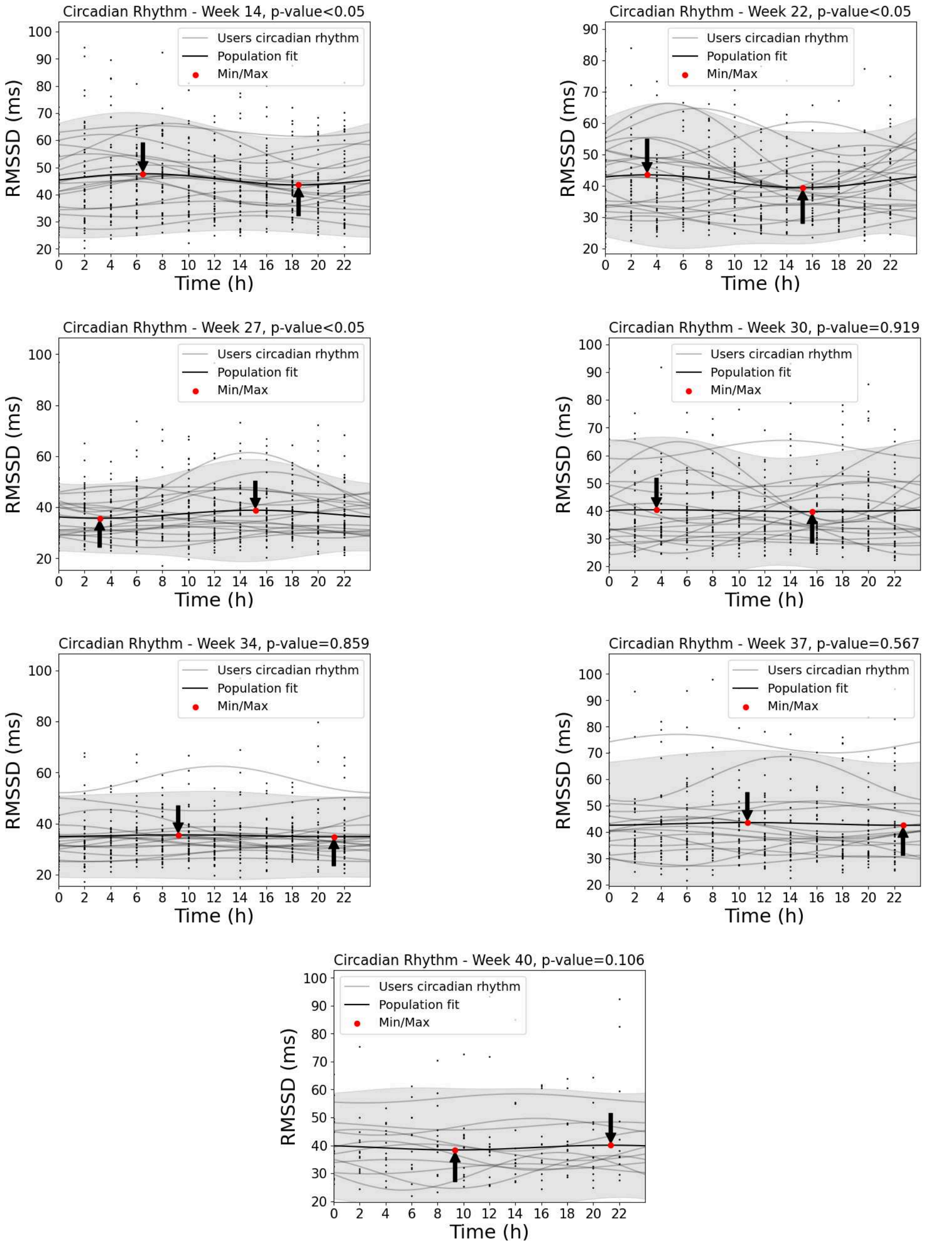

Figure 5: Cosinor analysis of the circadian rhythm of HRV across pregnancy weeks 14, 22, 27, 30, 34, 37, and 40. Each subfigure shows the circadian pattern of HRV for the corresponding pregnancy week. Individual participants' weekly-averaged circadian data and rhythms are represented by black dots and gray lines, respectively, while the bold black line depicts the population-mean HRV circadian rhythm. It should be noted that the number of data points (black dots) varies across weeks due to the unavailability of data from certain participants during specific weeks.

In week 27, the pattern was inverted compared to previous weeks, with the minimum RMSSD occurring at around 3:00 (below 40 ms) and the maximum observed slightly after 15:00 ($\sim$40 ms).

For weeks 30, 34, 37, and 40, the analysis did not reveal significant circadian variations, as both the maximum and minimum RMSSD values hovered around 40 ms. No notable differences were detected between day and night for the population-mean model during these weeks, indicating a less prominent oscillatory pattern.

## Discussion

Our study provides the first comprehensive investigation of HR and HRV circadian rhythms throughout pregnancy using continuous monitoring in free-living conditions. The key findings demonstrate: (1) the presence of significant circadian patterns in both HR and HRV throughout pregnancy, (2) systematic changes in these patterns across gestational weeks, particularly around week 27, and (3) distinct trimester-specific variations in rhythm parameters. These findings are particularly relevant given that the ANS plays a crucial role in regulating bodily functions and maintaining homeostasis during pregnancy, as it adapts to meet fetal requirements while protecting maternal health [1, 2]. While previous studies have documented circadian rhythms in various physiological parameters, including HR and HRV in non-pregnant populations [4, 5], the examination of these rhythms during pregnancy has been limited due to methodological challenges in longitudinal data collection and analysis of missing data. Our study addresses these limitations through the application of wearable technology and advanced data imputation techniques.

The findings of our study indicate a significant increasing trend in HR MESOR from week 15 to week 34, with the highest HR MESOR observed at week 34, followed by a pronounced decreasing pattern through the remainder of pregnancy (Figure 2c). In contrast, HRV MESOR shows a significant decline throughout pregnancy, suggesting higher levels of physiological stress up until week 34 (Figure 4c). This declining HRV trend can be attributed to the compensatory increase in heart rate required to meet the elevated cardiac output (CO) demands [35, 36], as well as the rising stress levels during pregnancy [37]. To further isolate the effects of stress on the ANS from the changes in HR due to increased CO demands, we analyzed circadian rhythm of normalized HRV metrics [38, 39], which confirmed the same pattern. As a result, we conclude that the HRV decrease is due to the ANS stress, which peaks around week 34, shown in (Figure 4c). After week 34, HRV MESOR increases sharply until the end of pregnancy. Given that MESOR represents the rhythm-adjusted mean for both HR and HRV, their patterns align closely with the mean HR and HRV reported in other studies, such as studies by Rowan et al. [29] and sarhaddi et al. [40]. However, an important distinction from the study by Rowan et al. [29] is that while they focused specifically on resting HR, which is typically lower, we measured HR throughout the entire day, resulting in higher values. Our approach provides the advantage of assessing HR and HRV during various daily activities and stressors, while other studies often rely solely on resting data.

The acrophase, described as the time of the amplitude/peak of HR, overall shows a significant delaying trend from the beginning of the second trimester (T2) to the end of pregnancy (Figure 2d), despite small fluctuations. This overall trend suggests that the time at which the maximum HR occurs shifts progressively later in the day as pregnancy progresses. The HR acrophase reaches its highest point in week 18 and its lowest point in week 22. However, a more detailed examination of HR acrophase throughout pregnancy reveals a more complex pattern.

The HRV acrophase shows greater fluctuations after week 24 (Figure 4d). An intriguing finding emerges in week 27, where the lowest HRV, which typically occurs in the evening due to daily stress, shifts to become the peak, suggesting a less stressful period during that time of day (Figure 5). Conversely, the minimum HRV occurs around 3 a.m. in week 27, a time traditionally associated with reduced stress due to sleep and rest (Figure 5). This reversed plot suggests increased stress levels during sleep, which may contribute to or result from sleep disturbances. These findings align with previous research on the declining sleep quality, as measured by the Pittsburgh Sleep Quality Index (PSQI) [41, 42], which begins to decline at the start of the third trimester compared to the second, despite a significant increase in total sleep duration [43]. This may explain why restless nights contribute to increased daytime fatigue and a greater need for afternoon naps. Indeed, a study shows a decrease in average nighttime sleep, an increase in the number of nighttime awakenings during months 7 and 8, and, finally, a higher frequency of daytime naps per week in month 8 and beyond compared to the average for the entire pregnancy [44]. These sleep disturbances are attributed to factors such as reflux, leg cramps, frequent urination, back pain, hip/pelvic discomfort, difficulty finding a comfortable position, baby movements, contractions, and concerns about delivery, all of which become significantly more common during the third trimester compared to the second [44].

The HRV amplitude, defined as the difference between peak and trough (highest and lowest values, respectively), shows an overall decreasing trend throughout the second and third trimesters (Figure 4b), indicating that the circadian rhythm of HRV becomes flatter as pregnancy progresses. In certain weeks, such as week 30 (Figure 4b and 5), the amplitude is particularly low, making it challenging to visually distinguish peaks and troughs. This reduced curvature in the HRV circadian rhythm can be attributed to an overall increase in stress levels compared to the early weeks of the second trimester, particularly elevated nighttime stress in later weeks, as the circadian rhythms in Figure 5 show more pronounced changes in the nighttime curve. These stress-related factors lead to reduced variation in RMSSD, often as a result of sleep disturbances. This suggests that elevated stress levels disproportionately affect nighttime and sleep more than daytime during weeks with low amplitude, as shown in the circadian rhythms in Figure 5.

Conversely, HR amplitude decreases from week 14 to week 27, after which it begins to rise toward the end of pregnancy (Figure 2b). In week 27, the lowest amplitude is observed, with minimal variation between daytime and nighttime

HR (Figure 3), which can be attributed to the influence of sleep disturbances. Additionally, as mentioned earlier, the HRV acrophase (timing of HRV amplitude) in week 27 indicates that the HRV trough occurs during nighttime, based on the circadian rhythms in Figure 4d, resulting in a reversed circadian pattern. While week 27 may not be the most stressful week of pregnancy, it exhibits the least HR variation (Figure 2b and 3), with the HRV trough occurring at night.

In conclusion, our study reveals changing patterns of HR and HRV, characterized by circadian rhythm parameters, with a level of detail not previously explored. The use of wearable health monitoring devices for continuous, non-invasive, and cost-effective data collection, combined with machine learning techniques, enabled enhanced signal processing, data imputation, and analysis of circadian rhythms. Integrating these findings into prenatal care can optimize clinical decision-making and treatment strategies by enabling personalized interventions that account for the timing of physiological changes, ultimately improving maternal and fetal outcomes while lowering healthcare costs.

Although this study yielded significant findings, this study has several limitations that should be addressed in future research. First, participants were all white women, and the geographical location of the recruitment site (Turku, Finland) limited participant diversity. Second, the study location experiences significant seasonal variations in daylight hours, ranging from approximately 19 hours in summer months (May-August) to 6 hours in winter months (December-February). As light exposure is a key photic cue for circadian rhythms, these variations may have influenced our results, particularly since participant recruitment occurred across different seasons. Third, physical activity levels, which can significantly impact both HR and HRV, were not controlled for or included in our analysis. Fourth, we did not account for participants' work schedules or differentiate between weekday and weekend patterns, which could have revealed important variations in circadian rhythms based on daily routines and social timing. Lastly, The sample size (n=30) provided limited data density, particularly during early (week 14) and late pregnancy (week 40), due to factors such as device familiarization period, late enrollment, and term deliveries before week 40.

While our study lays the groundwork for detecting abnormal circadian patterns in HR and HRV in pregnant women and their associations with pregnancy complications by first establishing normative patterns, future research should prioritize further investigation of HR and HRV circadian rhythms in women experiencing complications such as preterm birth and pre-eclampsia. Ultimately, developing a machine learning model to predict pregnancy complications by identifying deviations from normal circadian rhythms and recognizing similarities to patterns observed in women with complications will be crucial. Additional research directions include expanding these investigations to the pre- and post-partum periods, as well as exploring rhythm differences in pregnant women from diverse racial backgrounds and geographical locations with varying daylight exposure. Since circadian rhythms are closely linked with sleep [45], and our findings indicate patterns of high stress levels during nighttime, which may both result from and contribute to sleep disturbances, considering sleep parameters could provide valuable insights into circadian changes throughout pregnancy.

## Method

### Study Design

We investigated the circadian rhythm of HR and HRV through a longitudinal health monitoring study on pregnant women [32]. This observational study was conducted in free-living conditions with a sample of recruited pregnant women in Southwest Finland. In this study, participants were asked to wear Samsung Gear Sport smartwatches [34] to continuously record objective HR and HRV data during their daily activities. Additionally, a cross-platform mobile application was developed to gather subjective data such as Ecological Momentary Assessment (EMA) and background information using self-report questionnaires. Data collection spanned from gestational weeks 12 to 15 and continued for three months postpartum.

### Participants and Recruitment

Recruitment occurred from January 2019 to March 2020 in the Southwest Finland region. It included 30 pregnant women with previous full-term births and no history of pregnancy loss. Inclusion criteria for the study were singleton pregnancies, being at least 18 years old, proficiency in the Finnish language, and ownership of an Android or iOS smartphone.

Eligible participants were informed about the study objectives during face-to-face meetings. They were provided written informed consent and received study instructions and a smartwatch. Participants were instructed to continuously wear the smartwatch during pregnancy and three months postpartum. In this study, we utilized data from the low-risk group during the pregnancy period. Background information for the 30 participants in the low-risk group presented in Table 1.

### Research Ethics

This study was approved by the Ethics Committee of the Hospital District of Southwest Finland (Approval Number: Dnro 1/1801/2018). All study methods were designed and conducted in accordance with relevant guidelines, regulations, and protocols approved by the ethics committee. Written informed consent was obtained from all participants prior to their participation, agreeing to the publication of results with assurances of data de-identification to protect their privacy.

### Data Collection

HR and HRV data were collected from each participant using Samsung Gear Sport smartwatches [34]. These water-resistant, lightweight devices are capable of continuously recording PPG signals in everyday conditions. The smartwatches run on the open-source Tizen operating system, allowing for the devel-

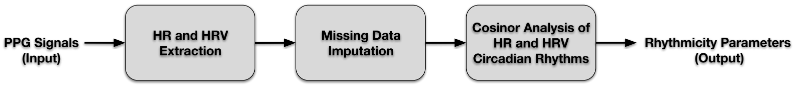

Figure 6: Data Analysis Workflow opment of customized applications for data collection and transmission.

PPG signals were recorded for 12 minutes every second hour at a sampling frequency of 20 Hz using customized applications installed on the smartwatches. This setup allowed effective data collection over approximately 2 to 3 days with satisfactory battery performance. We developed a custom application to transfer recorded data from the smartwatch to a cloud server via a Wi-Fi connection. Although the smartwatch's internal storage could hold data for up to two months, participants were instructed to upload their data at least once a week to ensure regular updates. On average, the PPG dataset used in this study included 111 minutes of recorded signal per day per participant.

### Data Analysis

#### Overview

We utilized the collected longitudinal PPG data to analyze the circadian rhythm of HR and HRV during pregnancy. Our data analysis consists of three key steps illustrated in Figure 6. First, we employed a reliable machine learning-based PPG processing pipeline to extract accurate HR and HRV data from raw PPG signals. Second, we leveraged a machine learning approach to impute any missing HR and HRV samples in data. Finally, we analyzed the circadian rhythm of HR and HRV during pregnancy using the Cosinor method.

#### HR and HRV Extraction

We extracted reliable HR and HRV parameters from longitudinal PPG signals from pregnancy using a PPG processing pipeline. PPG signals are highly susceptible to motion artifacts and noise, particularly when recorded in free-living conditions [46]. Corrupted PPG signals can lead to inaccurate HR and HRV measurements. To ensure the reliable extraction of these parameters from PPG, we employed a robust PPG processing pipeline, as proposed and evaluated in previous work [47].

The pipeline comprises multiple stages: filtering to remove unwanted frequencies, PPG signal quality assessment [48] to identify high-quality signal segments, PPG reconstruction [49] to correct minor noise and artifacts, and a robust PPG peak detection method [50]. Using this pipeline, we accurately extracted HR and Root Mean Square of Successive Differences (RMSSD), a key HRV metric, from 1-minute PPG signal segments.

The 1-minute window length was chosen due to the high susceptibility of PPG signals to noise while ensuring reliable data extraction in shorter segments.

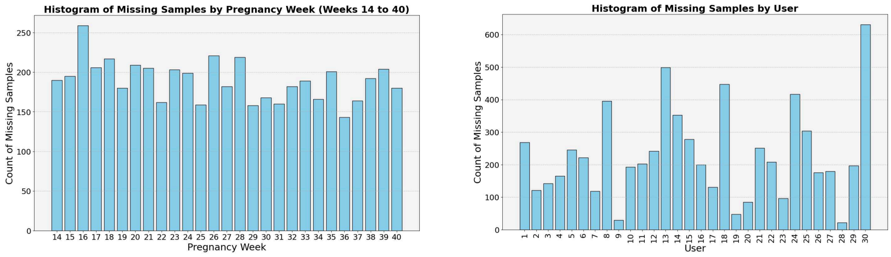

(a) (b)

Figure 7: Histogram of Missing HR/HRV Samples in Dataset by (a) Pregnancy Week and (b) User ID

HR and HRV values derived from the same 12-minute signal segment were averaged for further analysis. It should be noted that our analysis included data from days with HR and HRV samples from at least seven different hours.

#### Missing Data Imputation

We addressed the issue of missing HR and HRV samples in our dataset. As our data collection was conducted in free-living conditions, it is common to encounter missing data due to the user's hand movements or environmental noise. Since we included only the days with samples available from at least seven different hours and our data collection was conducted every two hours, the maximum number of missing values per day for a 24-hour rhythm was up to five. The distribution of missing HR and HRV samples by pregnancy week and user ID are demonstrated in Figures 7a and 7b. As indicated, the number of missing samples varied per user, likely reflecting differences in lifestyles and physical activity levels.

We imputed the missing HR and HRV samples using a Long Short-Term Memory (LSTM) network, a type of Recurrent Neural Networks (RNN) widely used for time-series data prediction [51]. LSTM networks capture and leverage temporal dependencies and patterns using memory cells that store information across time steps. These cells enable the network to preserve relevant information from previous samples, which is essential for predicting future samples in time-series data.

Our missing data imputation model consisted of three layers: one LSTM layer with 50 units and two dense layers. We trained and tested the model using sequential data created from available consecutive HR and HRV samples with a sequence length of six. The sequential data was split into training and testing sets, with 80% allocated for training. The model was trained over 100 epochs with a batch size of 5, using the Early Stopping technique to prevent overfitting. It is important to note that we normalized the sequential data by scaling it to a range of [0, 1] before training the model.

#### Cosinor Analysis of HR and HRV Circadian Rhythms

We employed Cosinor rhythmometry analysis [25] to explore the circadian rhythms of HR and HRV during pregnancy. Cosinor analysis is a common method for studying rhythmic data and has been widely used for analyzing circadian rhythms [52, 53, 54, 55]. This method involves fitting one or more cosine curves to rhythmic data using trigonometric regression models. A single-component Cosinor model is defined as follows:

$$Y(t) = M + A\cos\left(\frac{2\pi t}{\tau} + \phi\right) + e(t) \tag{1}$$

Where M is the Midline Statistic Of Rhythm (MESOR), A is the amplitude (half the distance between the peak and trough of the rhythm), $\tau$ is the period (duration of the rhythm), $\phi$ is the acrophase (the time point in the cycle where the peak occurs), and $e(t)$ is the error term. For multiple-component Cosinor analysis, which includes multiple cosine curves, the model is described by the following equation:

$$Y(t) = M + \sum_{i=1}^{N}\left(A_i\cos\left(\frac{2\pi t}{\tau_i} + \phi_i\right)\right) + e(t) \tag{2}$$

Where N is the number of components (cosine curves) in the model, and each component has its amplitude $A_i$, period $\tau_i$, and acrophase $\phi_i$.

We applied the Cosinor model with different numbers of components (1, 2, and 3). The best-fitting model was selected using the extra sum-of-squares F-test, a statistical method for comparing the goodness of fit for models with varying complexity.

We employed a population-mean Cosinor approach to analyze the HR and HRV circadian rhythms during pregnancy. Within each week, we had multiple 24-hour HR/HRV samples from a participant across several days. Our aim was to examine the weekly circadian rhythms for all participants throughout pregnancy. While the data from different participants were independent, the repeated measures within a week for the same participant were dependent. To address this structure, we applied the population-mean Cosinor analysis, as described in the literature [25, 56].

In this approach, a Cosinor model was first fitted to each participant's daily 24-hour HR and HRV data. Then, weekly averages for each participant were calculated by averaging the daily Cosinor results across several days of the week. Finally, the overall population rhythmic pattern for each week was determined by calculating the mean of the individual participants' weekly averages.

We implemented our analysis methods using the CosinorPy Python package [56], which is specifically designed for Cosinor-based rhythmometry. The analysis was conducted on a Windows machine equipped with an 11th Gen Intel Core™ i7 processor and 16 GB of RAM.

### Data Availability

The dataset utilized in this study contains sensitive health information, and the study informed consent form signed by participants prohibits the public release of the dataset due to ethics compliance. According to the current approval protocol from the Ethics Committee of the Hospital District of Southwest Finland, participants have been granted permission to use the collected data only for the purposes outlined in the consent form. Requests for data access are subject to individual consent and require a revision to the ethics committee's approved protocol. Researchers interested in accessing the data should contact the research project's principal investigator, Associate Professor Anna Axelin, at the Department of Nursing Science, University of Turku, Finland; email: anmaax@utu.fi.

### Code Availability

The developed code for this study is available from the corresponding author (M.R.) upon reasonable request.

## Acknowledgements

This research was supported by the Academy of Finland through the SLIM Project (grant numbers 316810 and 316811) and the U.S. National Science Foundation through the UNITE Project (grant number SCC CNS-1831918). The funding agency did not contribute to designing the study, collecting data, analyzing and interpreting data, or preparing this manuscript. Author SH declares that he was supported by the National Institute of Health grants (K99AG083234 and R01AG083799). Others did not receive any specific grant from funding agencies in the public, commercial, or not-for-profit sectors.

## Author Contributions

MR designed the study, performed data analysis, and drafted the manuscript; MF performed data curation, data analysis, and drafted the manuscript; IA contributed to study design, data collection, data analysis, and played a major role in manuscript revision; SH contributed to manuscript revision; FS contributed in data collection; HNV and AA were major contributors to participants' recruitment and manuscript revision; and PL and AR contributed to study design, data collection, data analysis and assisted in manuscript revision. All authors have read and approved the final manuscript.

## Competing Interests

Author SH reports receiving consulting fees from Achaemenid LLC, which are unrelated to this project, and declares no non-financial competing interests. All other authors declare no competing financial or non-financial interests.

## Appendix

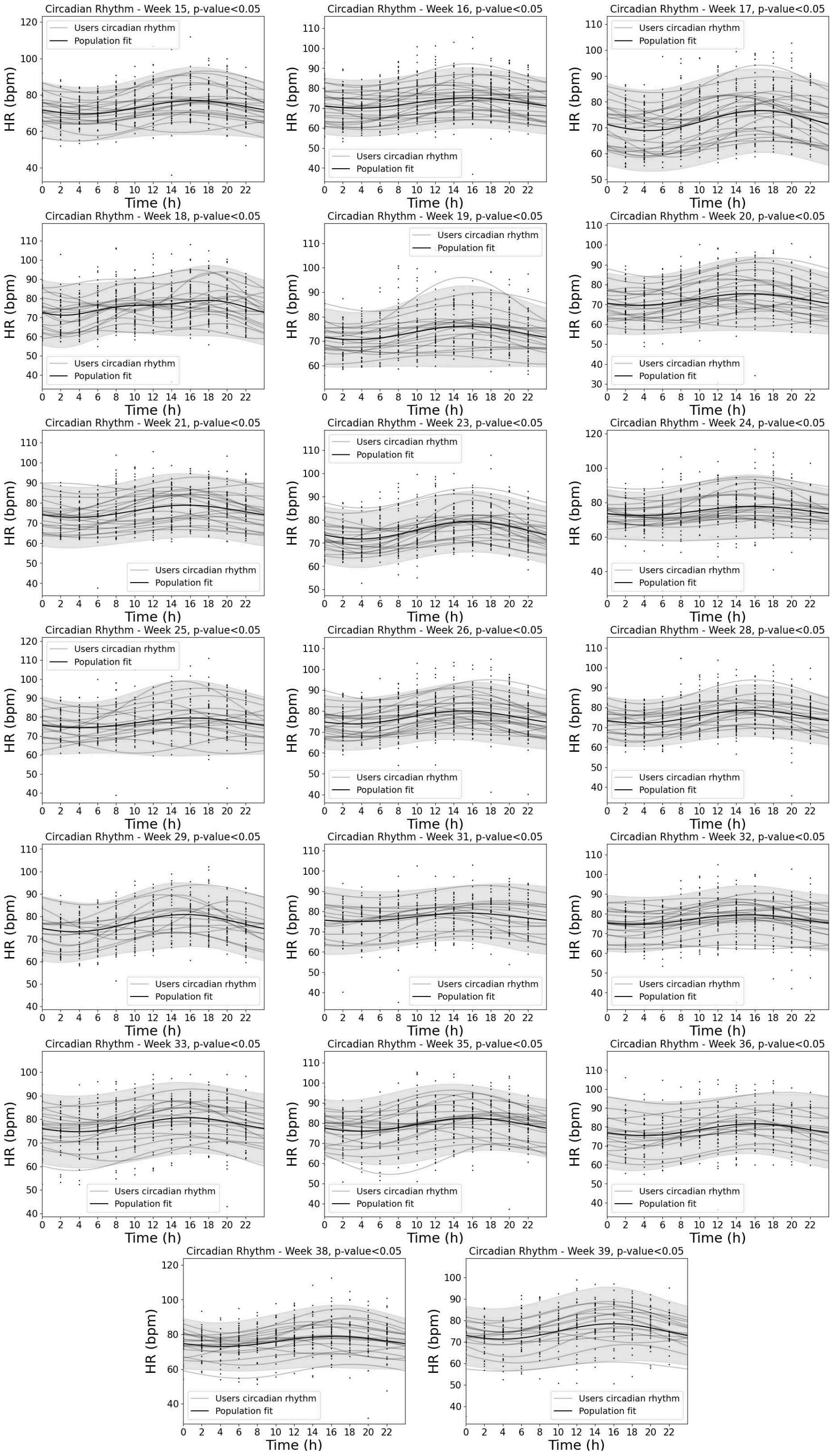

Figure 8: Cosinor analysis of the circadian rhythm of HR across pregnancy weeks. Each subfigure shows the circadian pattern of HR for the corresponding pregnancy week. Individual participants' weekly-averaged circadian data and rhythms are represented by black dots and gray lines, respectively, while the bold black line depicts the population-mean HR circadian rhythm. It should be noted that the number of data points (black dots) varies across weeks due to the unavailability of data from certain participants during specific weeks.

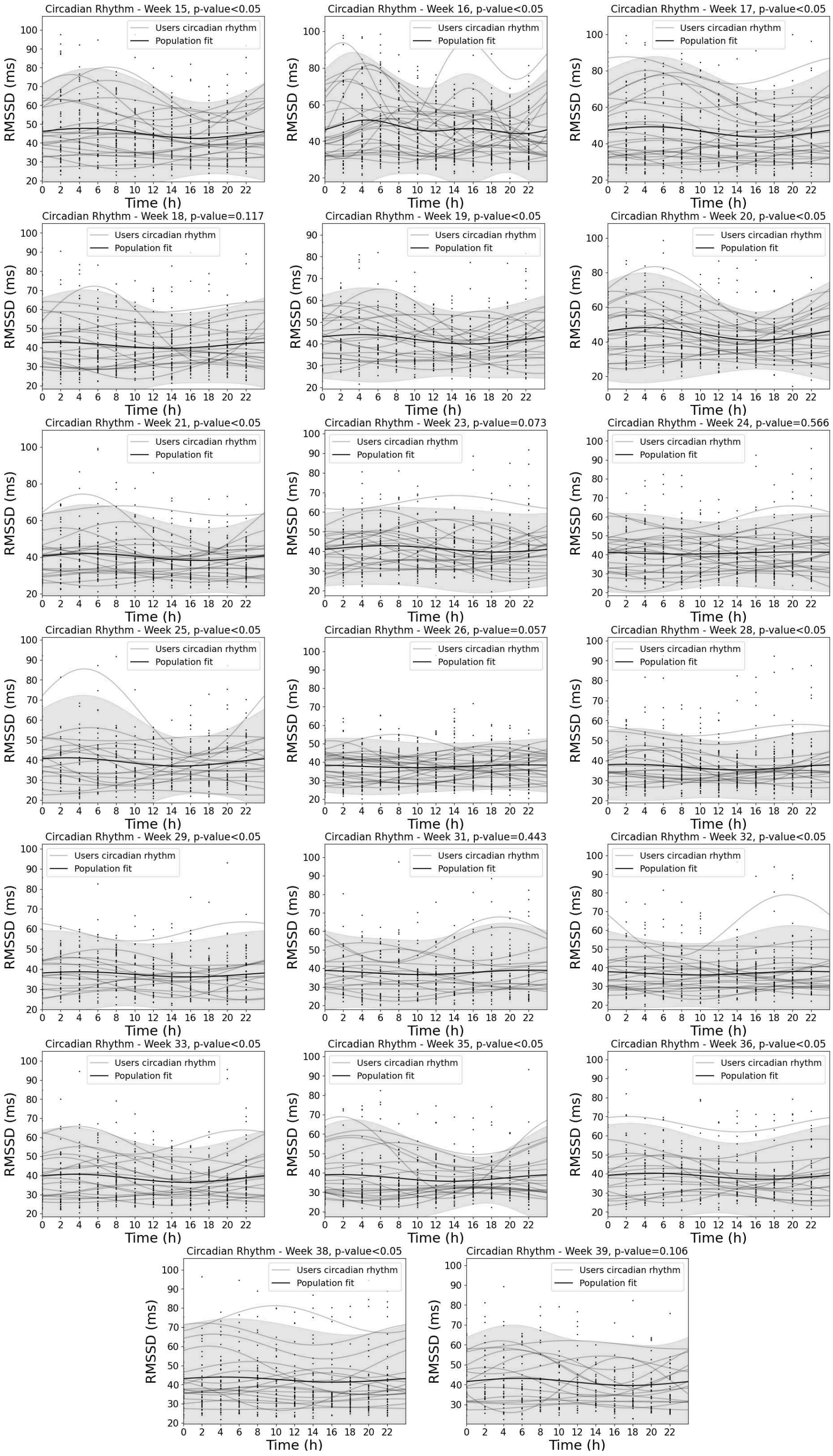

Figure 9: Cosinor analysis of the circadian rhythm of HRV across pregnancy weeks. Each subfigure shows the circadian pattern of HRV for the corresponding pregnancy week. Individual participants' weekly-averaged circadian data and rhythms are represented by black dots and gray lines, respectively, while the bold black line depicts the population-mean HRV circadian rhythm. It should be noted that the number of data points (black dots) varies across weeks due to the unavailability of data from certain participants during specific weeks.
